# An improved U-Net-based *in situ* root system phenotype segmentation method for plants

**DOI:** 10.3389/fpls.2023.1115713

**Published:** 2023-03-14

**Authors:** Yuan Li, Yunlian Huang, Mengxue Wang, Yafeng Zhao

**Affiliations:** School of Information and Computer Engineering, Northeast Forestry University, Harbin, China

**Keywords:** *in situ* root system, Minirhizotron method, U-Net, segmentation, transfer learning

## Abstract

The condition of plant root systems plays an important role in plant growth and development. The Minirhizotron method is an important tool to detect the dynamic growth and development of plant root systems. Currently, most researchers use manual methods or software to segment the root system for analysis and study. This method is time-consuming and requires a high level of operation. The complex background and variable environment in soils make traditional automated root system segmentation methods difficult to implement. Inspired by deep learning in medical imaging, which is used to segment pathological regions to help determine diseases, we propose a deep learning method for the root segmentation task. U-Net is chosen as the basis, and the encoder layer is replaced by the ResNet Block, which can reduce the training volume of the model and improve the feature utilization capability; the PSA module is added to the up-sampling part of U-Net to improve the segmentation accuracy of the object through multi-scale features and attention fusion; a new loss function is used to avoid the extreme imbalance and data imbalance problems of backgrounds such as root system and soil. After experimental comparison and analysis, the improved network demonstrates better performance. In the test set of the peanut root segmentation task, a pixel accuracy of 0.9917 and Intersection Over Union of 0.9548 were achieved, with an F1-score of 95.10. Finally, we used the Transfer Learning approach to conduct segmentation experiments on the corn *in situ* root system dataset. The experiments show that the improved network has a good learning effect and transferability.

## 1 Introduction

**Figure 1:**
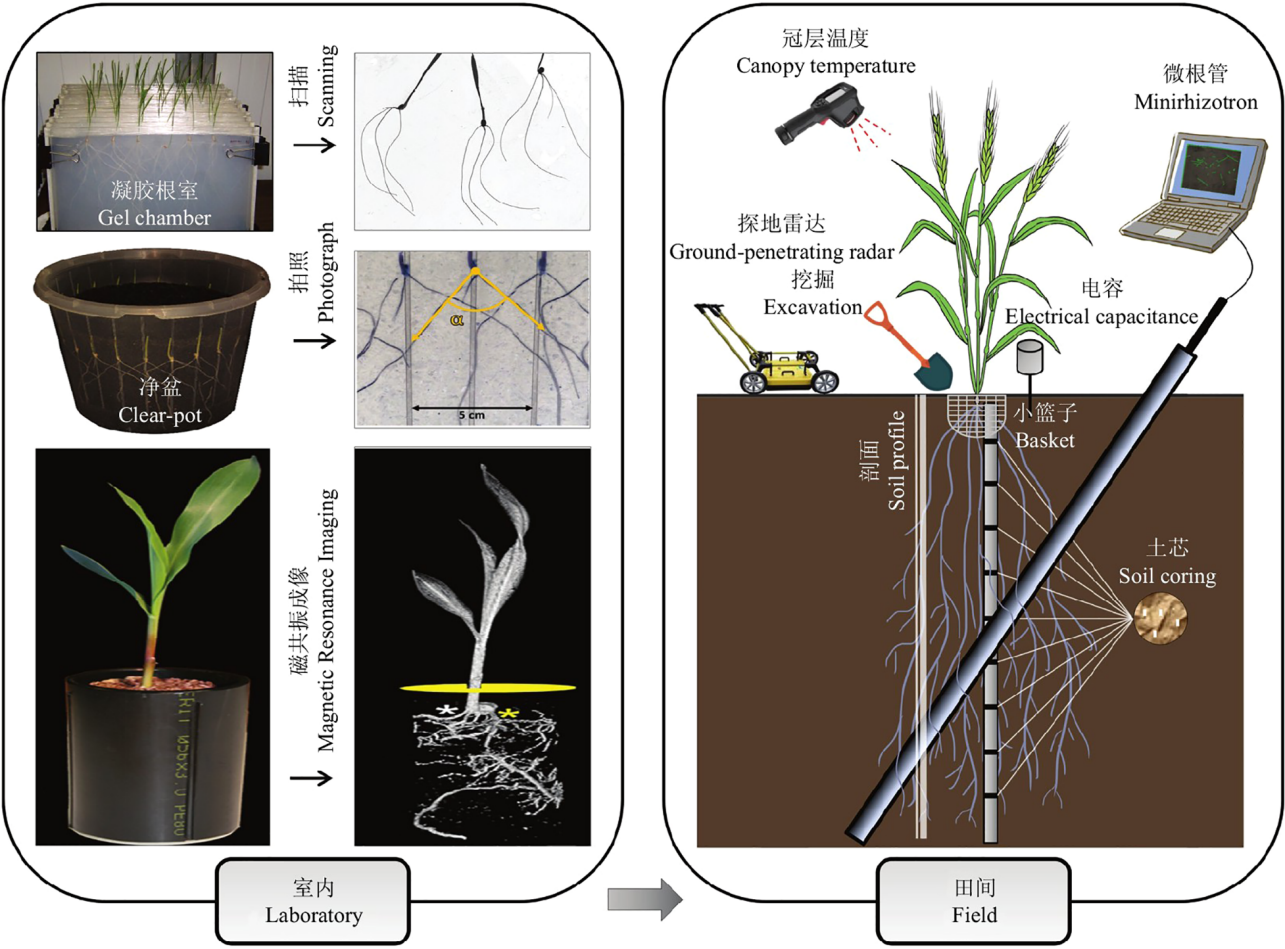
Main methods for studying root phenotypes.

The root system is an important part of the plant and is the main nutrient organ for plant growth and metabolism. Root morphological parameters are the main factors reflecting the growth status of the root system (Crush et al., 2010), and the growth status of the root can accurately reflect the health of the whole plant (Wei et al., 2015). Therefore, it is essential to study root phenology. Current root phenotyping methods are divided into indoor and field methods (see **Figure 1**) (Li et al., 2022). (1) The main methods used for indoor studies are the Gel Root Chamber method(Liu et al., 2013), the clear-pot method(Richard et al., 2015), X-ray computed tomography(CT)**(** Teramoto et al., 2020 **)**, and MRI (Van Dusschoten et al., 2016). Both the Gel Root Chamber and Net Pot methods use a soilless model and focus on plants with small root systems, which do not fully simulate the variable outdoor environment and are prone to bacterial infection. X-ray computed tomography and magnetic resonance imaging require excavation of the surrounding soil, and the rays will inevitably affect the roots to some extent, making them impossible to apply on a large scale for monitoring and research(Metzner et al., 2015; Rogers et al., 2016). (2) Research methods for field root studies are mainly divided into destructive identification methods and *in situ* identification methods. The destructive identification methods are Excavation(Zheng et al., 2020), Soil core(Wasson et al., 2014), Basket(Lou et al., 2015; Voss-Fels et al., 2018; Kitomi et al., 2020), Mesh bag, and Soil profile. The Excavation method is to observe the roots that are dug up and cleaned. The Soil Core Method involves drilling a smaller soil core than the root growth volume below the plant, then investigating indicators of root depth, biomass, and root length density in the core. The Basket method involves digging small baskets and investigating the number of roots in the pores at different locations. All these methods can cause damage to the roots, so they are not suitable when researching rare plants. With the development of technology, smarter and more efficient *in situ* identification methods have been explored, mainly including the Minirhizotron method (Bates, 1937), Ground Penetrating Radar method, and Capacitance method, which allow real-time monitoring of the root system. The Capacitance method is used to estimate root mass by reading the measured capacitance values. This method is often influenced by soil type, root type, and root development period. The Ground Penetrating Radar method uses an antenna to transmit and receive reflected electromagnetic waves, it is fast but only suitable for sandy soil(Alani and Lantini, 2020).

The Minirhizotron method is non-destructive, it has the feature of not affecting plant growth and is not affected by soil types. First, we bury the transparent tube bundle into the soil below the plant before sowing, and after the plant starts to grow, the root system will be attached near the tube wall, and then we feed the endoscope into the transparent tube bundle and scan the image of the root system on the tube wall, we can extract the root indicators from the image by observing the root system image. Svane et al. (2019) developed an automated microtubule monitoring platform, using the Videometer MR multispectral imaging system to obtain spectral images and the Videometer software to extract root images, which greatly improved the detection efficiency of the microtubule method. Wang et al. (2019) developed SegRoot software based on machine learning algorithms, which initially achieved automated separation of the root system and soil background in minirhizotron-scanning images. These technological innovations are driving the continuous improvement of the minirhizotron-scanning detection system and are expected to make it the preferred method for high-throughput *in situ* detection of root phenotypes in the field.

In conjunction with the development of deep learning, there have been practices and contributions to promote the point that automated separation of the root system from the soil background. In 2019, Smith et al. (2020) proposed the use of U-Net to segment collected chicory root images with an F1-score of 0.7. In 2020, Xu et al. (2020) explored and analyzed plant root image segmentation using U-Net network models of different depths based on Transfer Learning and semantic segmentation of U-Net networks, Shen et al. (2020) performed used the DeepLabv3+ method for segmentation tasks in homogeneous soils. In 2021, Kang et al. (2021) introduced a sub-pixel convolutional DeepLabv3+ semantic segmentation model for cotton root images by using a sub-pixel convolutional layer instead of the bilinear interpolation up-sampling approach and adding additional interpolation functions in the convolutional layer. Current methodological updates have led to pixel accuracy of more than 95% for the automatic separation effect of root systems from soil background. However, lighter and more transferable segmentation models still need to be further explored.

The main difficulties in root segmentation are as follows: (1) long training time for deep learning, difficult data collection, the influence of different soil backgrounds on the segmentation task, and difficulty in datasets with high generalizability. (2) Complex background, the extreme imbalance between the root system and the background, and the presence of small object interference. To address these issues, we improved the U-net. Firstly, we used ResNet50 (He et al., 2016a **)** as the backbone network, which allows better extraction of image features and access to higher-level semantic information, while preserving information lost at different levels. Secondly, we added the PSA attention PSA(Zhang et al., 2023) in the up-sampling process, this method enables the network to focus on features at different scales, and focusing on features at multiple scales can better segment the minutiae. Finally, to solve the problem of uneven root distribution in the dataset, the Dice-Focal loss (Gammoudi et al., 2022) hybrid loss function is used to further improve the training effect. In this paper, control experiments and transfer learning experiments are conducted to validate and compare the performance of the improved model on different datasets, proving that the improved network has good transferability and better learning ability.

## 2 Materials and methods

**Figure 2:**
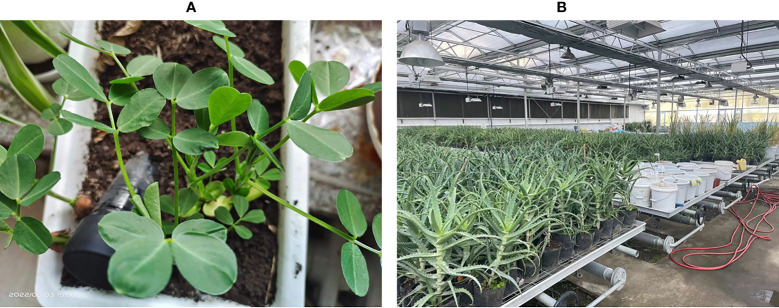
Experimental scenario. **(A)** Minirhizotron installation, **(B)** Greenhouse scenes.

The experiment was carried out in 2022 in the seedling laboratory of Northeast Forestry University in Harbin, China, in a temperate continental monsoon climate. The lab is equipped with a sophisticated monitoring system, control system, and irrigation equipment, which allow real-time monitoring of temperature, humidity, CO2 concentration, and light intensity in the room. See **Figure 2B** for the scenario.

### 2.1 Image collection

#### 2.1.1 Minirhizotron installation

The minirhizotron tube is made of acrylic and the tube diameter size is chosen to be 4cm, both ends of the tube are sealed. The part exposed above the soil is covered with opaque tape to prevent light from entering the tube, which does not interfere with plant growth and does not allow foreign objects to enter and cause observation problems. Seven months before starting the image collection, the tube is buried in the soil near the plant at a 45-degree angle until the plant roots have grown. The diagram of the equipment installation is shown in **Figure 2A**. In this paper, a total of 350 individual plants with 150 peanut plants and 200 corn plants were used to collect a total of 2000 images of peanut roots and1900 images of corn roots as data sets.

#### 2.1.2 Image acquisition and labeling

The image acquisition equipment uses a 4.9mm dual-lens endoscope which is connected to a computer for data storage, using the side camera of the endoscope near the inner wall. The original pixels of the images were 1920*1080 pixels and the images were saved in JPG format. After rejecting the unqualified images, the image annotation process is carried out.

**Figure 3:**
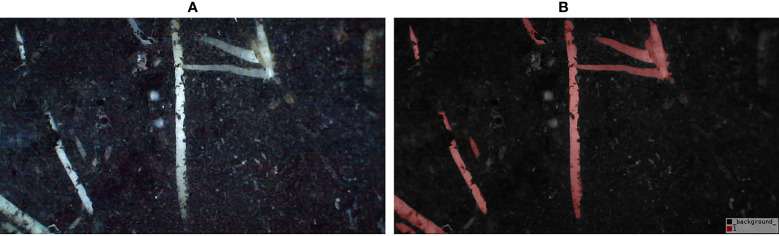
Image annotation map. **(A)** Photographs of the root system, **(B)** Sample of annotated image.

For image annotation, manual annotation was performed using the Lableme software (a graphical interface image annotation software, inspired by http://labelme.csail.mit.edu/. It is written in Python and uses PyQt for the graphical interface. It can annotate images as polygons, rectangles, circles, polylines, line segments, and points). All image labeling was carried out by an experienced teacher. The resulting label image has a root pixel value of 1 and a background pixel value of 0. The color scheme of the label is RGB=[128, 0, 0] for the root and RGB=[0, 0, 0] for the background, and the label is saved in png format. Each image takes about 10 minutes to annotate. The labels and labeled images are shown in **Figure 3**.

### 2.2 Model

#### 2.2.1 Segmentation model

U-Net(Ronneberger et al., 2015) was originally proposed as a solution to the problem of medical image segmentation, and as a whole is an Encoder-Decoder structure. The encoder part: two 3x3 convolutional layers (ReLU) + one 2x2 max-pooling layer form a down-sampling module, which consists of iterations of the down-sampling module. The number of channels is doubled with each down-sampling. The original thesis uses valid convolution (convolution starts when the filter is all inside the image), so for each valid convolution, the height and width of the feature map are reduced by 3-1 = 2 pixels respectively, as there is no padding. The decoder part: a 2x2 up-sampling convolutional layer (ReLU) + Concatenation (crop the feature map corresponding to the output of the left half and then add it to the up-sampling result of the right half) + two 3x3 convolutional layers (ReLU) iteratively, with the last layer turning the number of channels into the desired number of categories by a 1x1 convolution. After each up-sampling transpose convolution, the height and width are doubled, while the channel is halved and used for merging with the shallow feature map on the left. The main benefit is that the deeper the network layer, the larger the field of view of the feature map obtained. The shallow convolution focuses on texture features, while the deeper network focuses on the essential kind of features, so both deep and shallow features are meaningful. While each down-sampling refines the features, some edge features are inevitably lost, and the lost features are not recovered from the up-sampling. Through the stitching of features, a recovery of edge features can be achieved.

**Figure 4:**
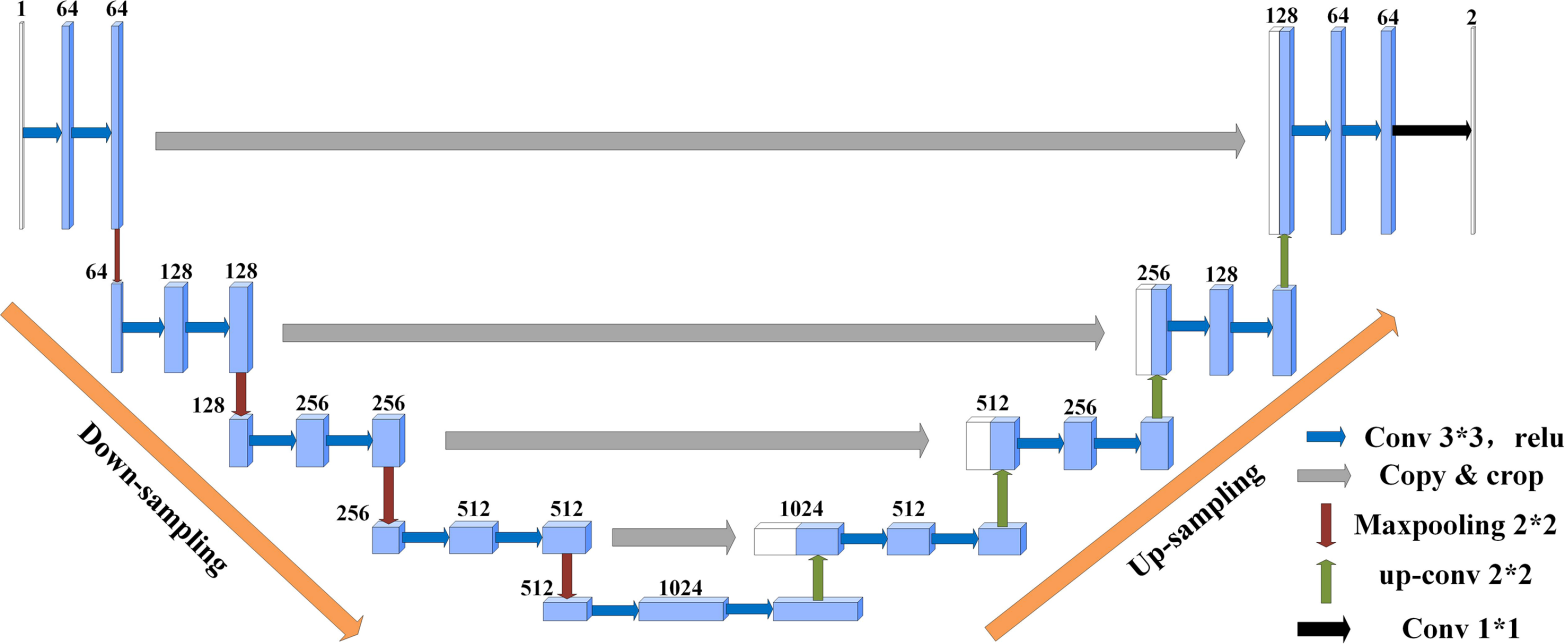
U-Net structure.

Suppose the initial image is 224x224, after feature extraction, there will be four different feature maps of 112x112, 56x56, 28x28, and 14x14. Then we up-sample or deconvolve the 14x14 feature map to get a 28x28 feature map, which is stitched with the previous 28x28 feature map, and then convolve and up-sample the stitched feature map to get a 56x56 feature map, which is then stitched with the previous 56x56 feature map, convolved, and up-sampled again. After four up-sampling, a prediction of 224x224 with the same size as the input image is obtained. The complete structure of U-Net is shown in **Figure 4**.

#### 2.2.2 Model improved

**Figure 5:**
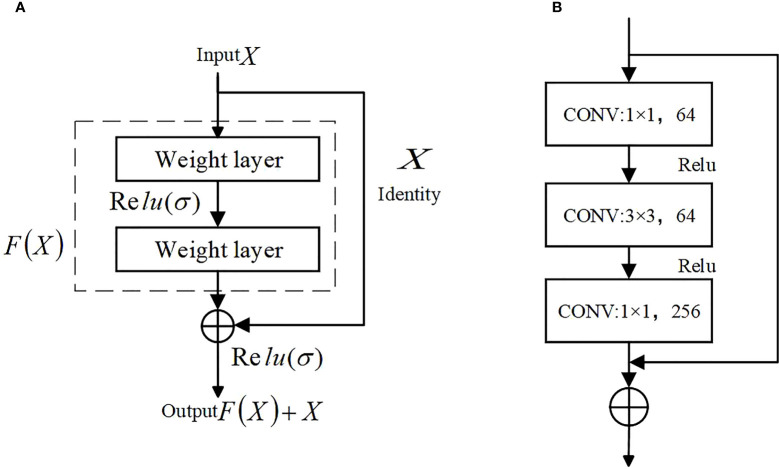
Residual structure. **(A)** Residual structure, **(B)** Bottleneck residual Block.

**(1)** The main role of the encoder part of U-Net is to extract features, the proposal of ResNet (Deep residual network)(He et al., 2016b) in 2016 is a milestone event in CNN computer image processing, which solves the problem of saturation, the decline in accuracy due to the deeper depth of the neural network and the deterioration of the network performance with the number of layers. Compared with the original structure, using the residual network as the main structure for feature extraction can better extract features and reduce the loss of features. The structure of the residual network is shown in **Figure 5**.

ResNet has two types of residual networks. The ResNet50 used in the paper belongs to the deep network constructed by Bottleneck residual Block (see b in **Figure 5**). It first undergoes 1*1 convolution for dimensionality reduction and 3*3 spatial convolution mainly used to extract image features. The 1*1 convolutional layer increases the non-linear capability of the network and improves its expressiveness.

**(2)** The attention mechanism was first proposed by Tsotsos et al. (1995) and applied in the field of visual images. In 2014, Mnih et al. (2014) applied it to a neural network RNN for image classification. In computer vision, the applied attention mechanisms are divided into three main blocks: (i) channel attention mechanism, (ii) spatial attention mechanism, and (iii) self-attention mechanism.

**Figure 6:**
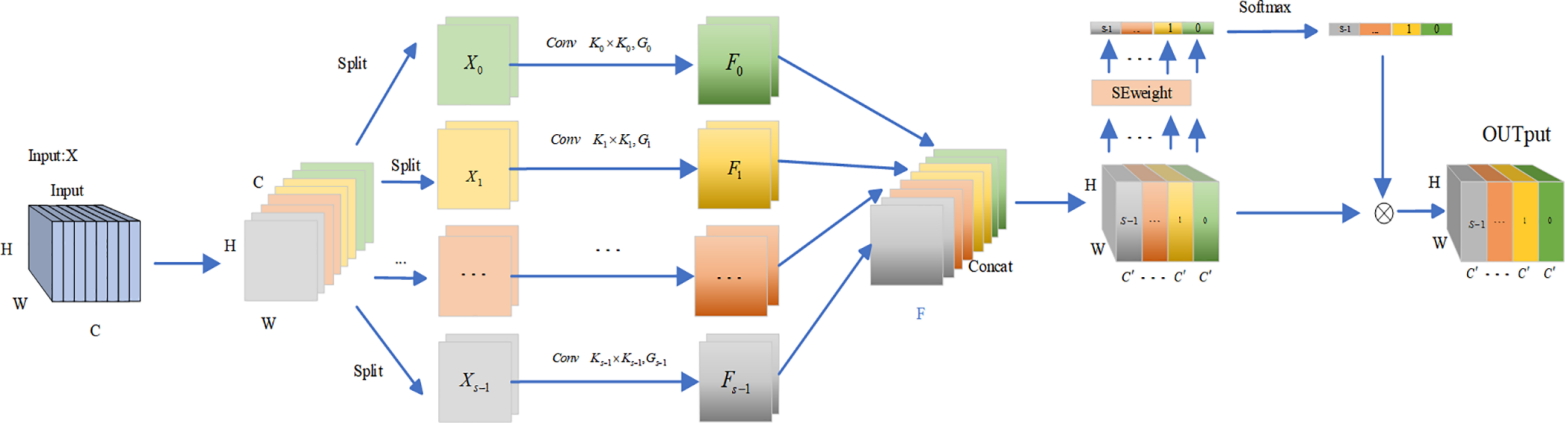
PSA attention structure.

Zhang et al. (2023) proposed a new backbone architecture called EPSANet, which uses a new module called Pyramid Split Attention (PSA), the PSA module offers low cost and high performance. First, the input feature maps are extracted to obtain multi-scale feature maps in the channel direction and cross-channel interaction is performed. Then the features at multiple scales are fused by the SE attention module(Hu et al., 2018). Finally, the weights of attention are applied to the corresponding feature maps by softmax to obtain feature maps with richer multi-scale feature information as the output. To allow the strong semantic information at the higher level to better guide the information at the lower level, we introduce the PSA module in the decoder part. the structure of the PSA module is shown in **Figure 6**.

As a plug-and-play attention mechanism module, PSA can maintain a relatively high resolution in the channel and spatial dimensions, resulting in less information loss. At the same time, to solve the problem of computational and memory explosion when modeling channels and spaces without dimensionality reduction, PSA uses polarization filtering, which is used to enhance or weaken features at each node. By introducing the PSA attention mechanism, a more fine-grained non-linear function can significantly improve feature utilization, which is reflected in the segmentation task in terms of greater refinement in edges and small regions, resulting in improved performance.

**(3)** In addition to using a more powerful backbone network for feature extraction and the PSA mechanism for better improving the feature processing power, a Dice-Focal loss function is used to address the problem of imbalance between the background and the target region.

In the dataset used, 80% of the images had an extreme imbalance between the root system and the background, with the root system occupying only a small portion of the whole image. Dice loss(Milletari et al., 2016) has good performance for scenarios with a severe imbalance between positive and negative samples, and the training process focuses more on the foreground region. However, using Dice loss alone has a negative impact on backpropagation and tends to make the training unstable. Focal loss(Lin et al., 2017) was originally used in the image field to solve the model performance problem caused by data imbalance. It can adaptively adjust the percentage of loss values for each pixel. The new loss function is:


$$L=L_{Dice}+L_{fl}=C-\sum_{c=0}^{C-1}\frac{TP_{p}\left(c\right)}{TP_{p}\left(c\right)+\alpha FN_{p\left(c\right)}+\beta FP_{p}\left(c\right)}-\frac{1}{N}\sum_{c=0}^{C-1}\sum_{n=1}^{N}g_{n}\left(c\right)\left(1-p_{n}\left(c\right)\right)$$

$$TP_{p}\left(c\right)=\sum_{n=1}^{N}p_{n}\left(c\right)g_{n}\left(c\right)$$

$$FN_{p}\left(c\right)=\sum_{n=1}^{N}\left(1-p_{n}\left(c\right)\right)g_{n}\left(c\right)$$

$$FP_{p}\left(c\right)=\sum_{n=1}^{N}p_{n}\left(c\right)\left(1-g_{n}\left(c\right)\right)$$

Among the above equations, *c* denotes the pixel class of the image; *TP_p_* (*c*), *FN_p_* (*c*), and *FP_p_* (*c*)) are the true positives, false negatives, and false positives of class c, respectively; *p_n_* (*c*) is the prediction rate of the nth pixel of class *c*; *g_n_* (*c*) is the nth expert annotation value of class *c*; *C*2 denotes the number of classes; *N* denotes the number of pixels in the training batch; *α* and *β* are the equilibrium false positives and false negatives coefficients.

**Figure 7:**
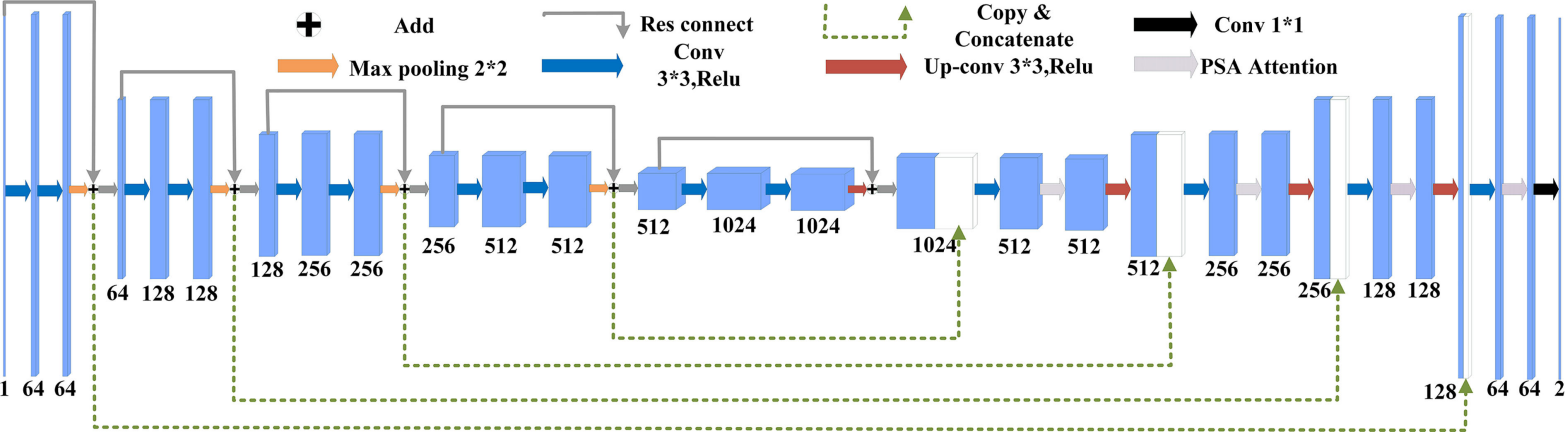
Complete structural diagram.

The complete structure of the improved diagram is shown in **Figure 7**.

### 2.3 Evaluation

To objectively evaluate the effectiveness of the model in the root segmentation task, three metrics, F1-score, pixel accuracy(*PA*), and Intersection Over Union(*IOU*), were taken to evaluate the model in this paper. The calculations are publicly shown below.

**Table:** 


Confusion matrix		Real	
		Positive	Negative
Predicted	Positive Negative	TP FN	FP TN


$$F1=2\cdot \frac{precision\cdot recall}{precision+recall}$$

$$PA=\frac{TP+TN}{TP+FP+FN+TN}$$

$$IOU=\frac{TP}{TP+FP+FN}$$

The *F*1 value is a combined assessment of both the *precise* and *recall* metrics, which can effectively reflect the overall effectiveness. Where *precision* indicates the percentage of all samples where the model predicted a positive case, and *recall* indicates what percentage of all samples with positive true labels were predicted.


$$precision(P)=\frac{TP}{TP+FP}$$

$$recall\;(R)=\frac{TP}{TP+FN}$$

Pixel accuracy(*PA*) represents the percentage of correctly predicted pixel values to the total pixel values, and Intersection Over Union represents (*IOU*) the ratio between the intersection and the concatenation of predicted results and true labels for a category.

## 3 Results

This section of the article contains three main parts of experiments. The first part of the experiment demonstrates the changes in each assessment metric before and after the model improvement, and this validation part of the dataset is all from self-collection. The second part conducts ablation experiments to demonstrate the validity of each improvement step. The third part validates the transferability of the model by taking a portion of the corn root data collected in Jinan, Shandong Province by the same group, the corn root data set will be annotated in the same way. The difference is that the soil characteristics of the two sites differ. The soil for peanut cultivation is meadow black soil and the soil for corn cultivation is tidal soil. In the experiments of peanut root segmentation, a total of 2000 images were used, and the training set, validation set, and test set were divided according to 8:1:1.

The experiments were conducted using a 64-bit Windows 10 operating system, NVIDIA GeForce RTX 3090 graphics card with 24GB of video memory, 14-core Intel(R) Xeon(R) Gold 6330 CPU at 2.00GHz, and 180GB of RAM. Python version 3.8.10 was used as the language and Pytorch version 1.8.0 was used as the development framework for deep learning. A total of 100 rounds of training were conducted using the Adam optimizer, with momentum set to 0.9 and the learning rate decreasing by cosine annealing. In the training model using root images, a mixture of dice loss and focal loss was chosen as the loss function, the batch size was set to 28, a total of 100 epochs were trained, and the initial learning rate was set to 0.0001.

### 3.1 Improved model analysis

**Figure 8:**
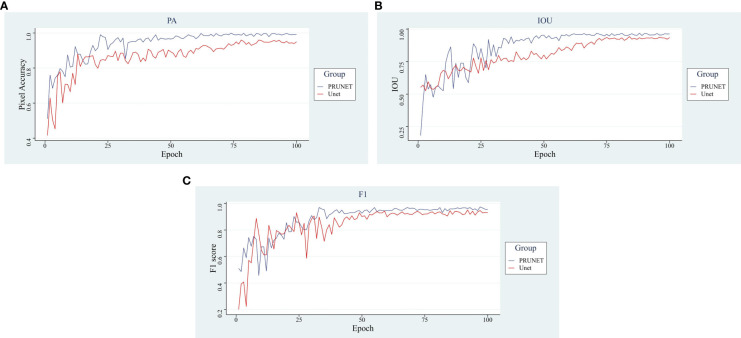
Analysis of main indicators. **(A)** Pixel Accuracy, **(B)** Intersection Over Union, **(C)** F1-score.

In **Figure 8**, the main evaluation metrics of the network before and after the improvement are compared. The improved model shows faster convergence and better performance, reflecting a stronger deep-learning capability. In terms of the performance of the main evaluation parameters, both models show a jittering upward trend in the general trend, with three evaluation criteria of the improved model stabilizing after 55 rounds, while the pre-improved network only shows stability after 75 rounds. In addition, the improved model outperformed the pre-improved model in all three metrics. A comparison of the before and after improvement models is shown in **Table 1**.

**Table 1:** Comparison of evaluation parameters before and after improvement.

	PA	IOU	F1
U-Net	94.93	93.23	92.95
PRU-Net	99.17	95.48	95.10

**Figure 9:**
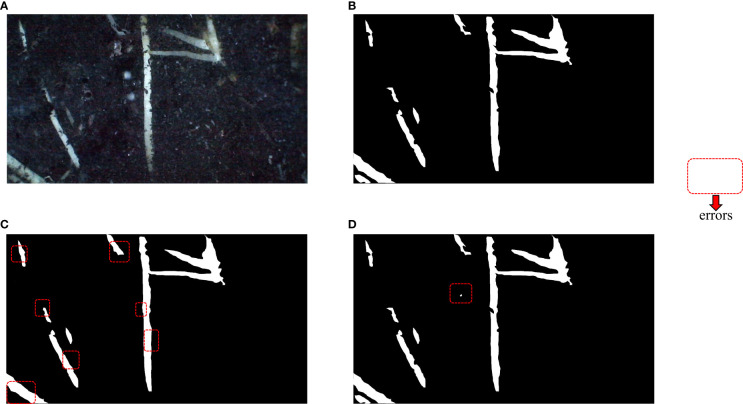
Visualization of segmentation effects. The red dashed boxes represent significant differences from the real mask. **(A)** In situ root system, **(B)** Labeled real mask map, **(C)** Unet segmentation effect display, **(D)** PRUnet segmentation effect display.

In terms of pixel accuracy, it reached over 99%, an increase of 4.24% accuracy relative to the improvement, a 2.25% increase in Intersection Over Union, and a 2.15% increase in the F1-score compared to the pre-improvement model higher, the improved model showed good segmentation performance. The visualization of the segmentation effect before and after the improvement is shown in **Figure 9**.

(Xu et al., 2022) improved Unet and conducted experiments on the hydroponically grown soybean seedling roots, the pixel accuracy of the experiments reached 99.64. (Lu et al., 2022) improved the U-net model to P-T-U-Net model (U-Net based on prior knowledge and transfer learning). Pixel accuracy (PA) of 97.7 and a mean F1-score of over 90 were achieved in segmenting the pepper roots. (Thesma and Mohammadpour Velni, 2023) produces realistic high-resolution root images with reduced pixel-level imbalance by cGAN. Experimental segmentation models on GAN-generated images yielded high pixel accuracy (over 99%). Compared with the latest papers mentioned above, the data used in this paper are all from real root images, which maximally simulate the actual growth environment of plants, our proposed method has experimented on different data sets, and the experimental results are much better.

**Figure 9** above mainly shows the segmentation effect of the segmentation method before the improvement and the segmentation method used in this paper. (A) shows the captured root system image, (B) shows the labeled mask image, (C) shows the segmentation effect of the U-Net network, and (D) shows the improved network segmentation effect. The red box represents a significant discrepancy from the real mask image, after visual comparison. The pre-improved network basically achieves an overall accurate segmentation, but often connects gaps at the fine edges. The improved network shows better segmentation in the fine edge but mistakes the background for the root system at one point in (D) in **Figure 9**. The visualization analysis demonstrates that the improved network does indeed perform better segmentation.

### 3.2 Ablation experiments

This section focuses on ablation experiments. The U-Net with only the improved backbone network ground is named R_U-Net, the U-Net with the PSA attention mechanism added is named P_U-Net, and the U-Net using the new loss function is named D_U-Net. Their main metrics are compared as shown in **Table 2**

**Table 2:** Ablation experiments.

	PA	IOU	F1
U-Net	94.93	93.23	92.95
R_U-Net	97.76	95.11	93.67
P_U-Net	96.41	95.02	94.88
D_U-Net	95.47	93.44	93.17

**Figure 10:**
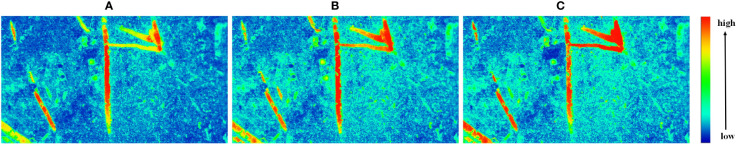
Feature extraction visualization. **(A)** Original network, **(B)** After replacing the backbone with ResNet, **(C)** After adding the PSA module.

The focus of the three improvements is different, U-Net is a typical encoder-decoder structure, and the backbone network part is mainly to extract features. To extract features better, we replace the original backbone network with ResNet. After using ResNet, Pixel accuracy (PA) is improved by 2.83%, Intersection Over Union represents (IOU) is improved by 1.88%, and F1-score is improved by 0.72%. **Figure 10** visualizes the change in feature extraction capability of the improved network.

In **Figure 10**, the closer the color is to the red part above, the deeper the feature is associated with the root. (A) shows the original U-Net network, and (B) shows the U-Net after replacing the backbone network, (C) indicates the addition of the PSA attention module. it is obvious that the effect of (A) in **Figure 10** on the root edge segmentation in the image is not obvious, but the effect of noise suppression in the image is more obvious. After improving the backbone network, the effect of extracting features related to the root system is stronger. The red part of the figure is significantly increased, and the edge contour part is more detailed.

The PSA mechanism module is added to the up-sampling section, mainly to improve its ability to utilize features and improve segmentation accuracy. With the addition of the PSA module alone, Pixel accuracy (PA) is improved by about 1.48%, Intersection Over Union represents (IOU) by about 1.79% and the F1-score to 94.88%. The positive optimization effect of the attention mechanism module on the model network is successfully demonstrated. In **Figure 10**, it can be seen that the addition of the PSA module has a positive optimization effect on the extraction of inconspicuous fine roots and more obvious features, making the extraction effect more obvious.

**Figure 11:**
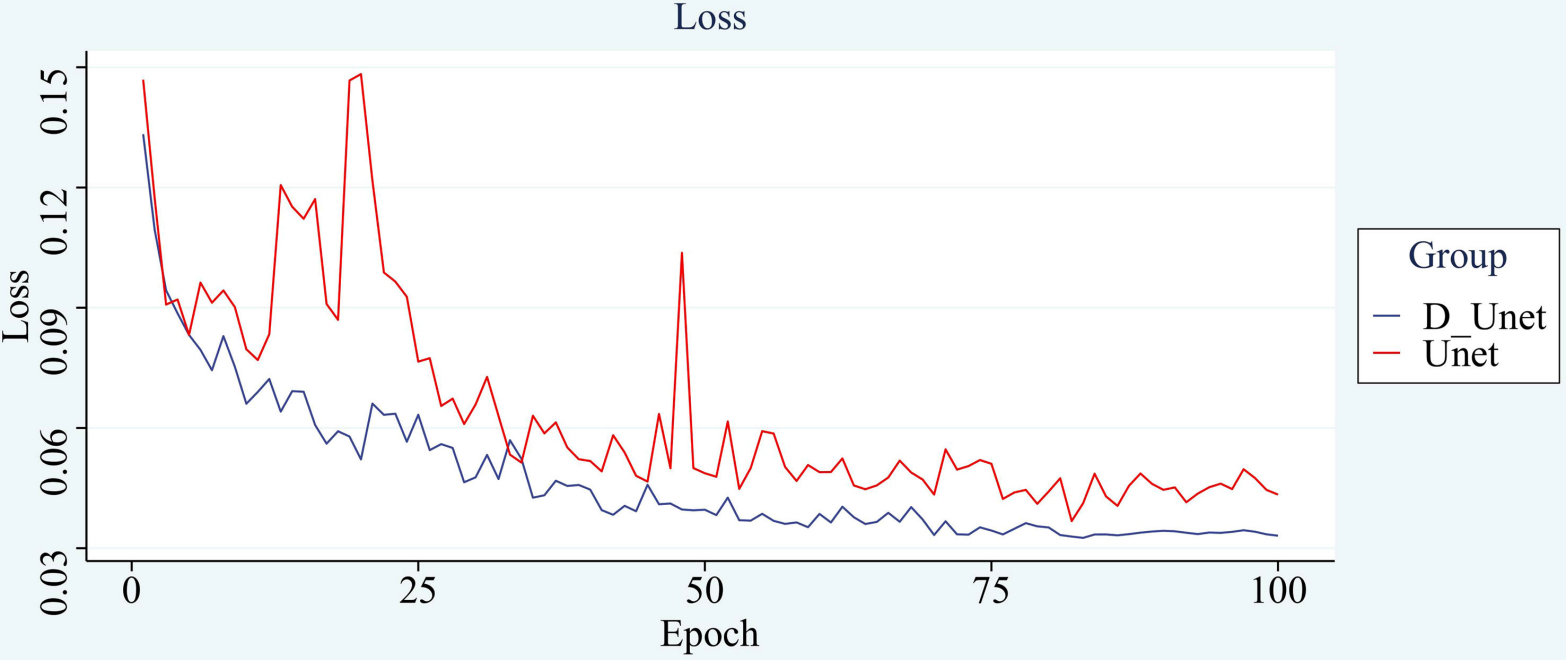
Loss comparison.

After using the new loss function, the improvement is small, but the speed of model convergence is found to be improved during the training process, and the number of rounds needed to be iterated is reduced with the same set of training hyperparameters for both networks. The original network shows a stable trend in loss around 75 rounds, and the improved network shows a stable trend after 68 rounds. The improved network is less volatile and shows more stable results. The comparison curve of its loss is shown in **Figure 11**.

The improved network outperforms the convergence speed of the original model. It also proves to a certain extent that the improved model has a more powerful learning ability and can accelerate the convergence speed. Its application is beneficial to the overall effect of the model.

### 3.3 Transfer learning analysis

**Figure 12:**
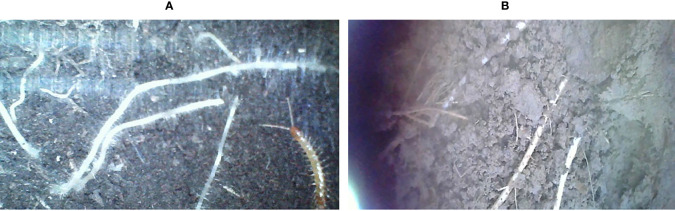
Partial data display. **(A)** Peanuts cultivated in black soil, **(B)** Corn cultured in tidal soil.

Transfer Learning is a hot problem in deep learning. To demonstrate the transferability of the model before and after improvement, another data set collected by our group in Jinan, Shandong Province is used for transfer learning in this section, with corn as the crop and tidal soil as the culture soil. Some pictures of the two datasets are compared as shown in **Figure 12**. The cultivated soil of peanuts is biased towards black, while the cultivated soil of corn is biased towards yellowish brown, which can clearly distinguish the difference between cultivated crops. At the same time, to explore the application strategy of the data, different amounts of training data were taken for training in this section, and the number of training sessions required for them to reach a stable effect was recorded.

**Figure 13:**
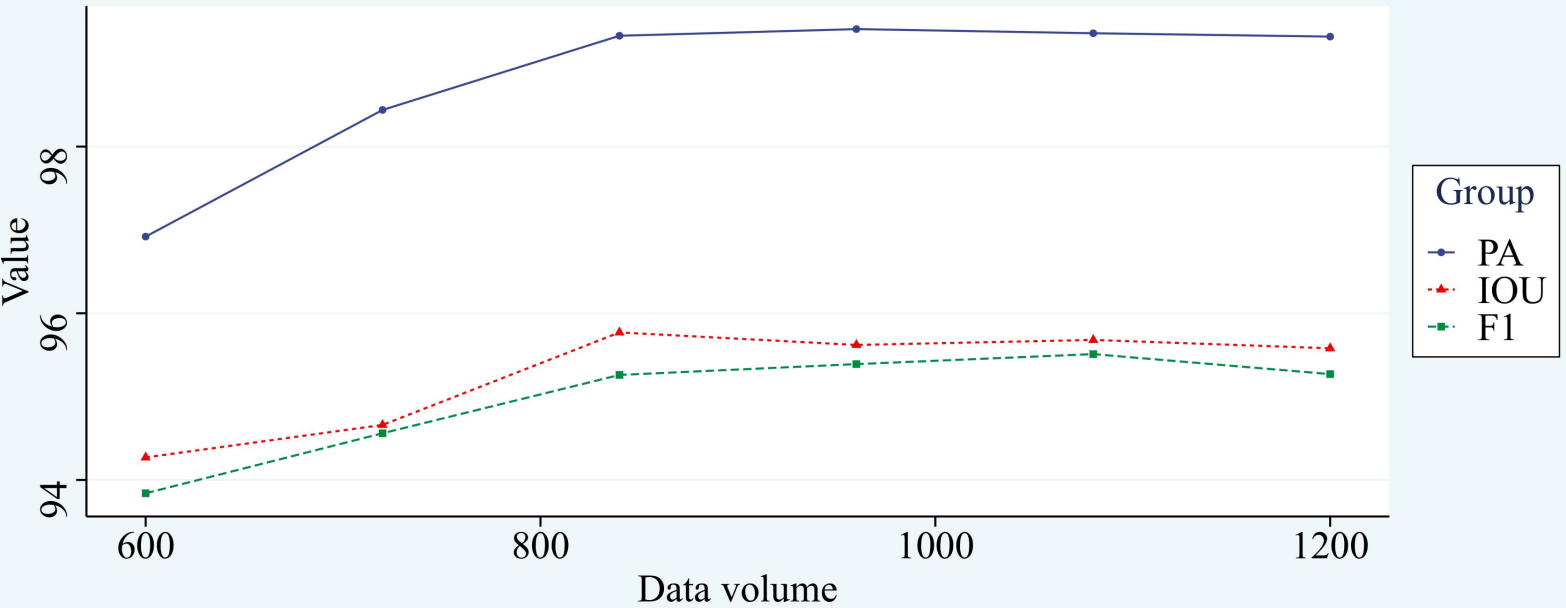
Data volume and training effect.

In general, under the premise of the same training effect, the fewer samples of training set required by the model represents the better performance of the model. The common division ratio of the training set and test set is 7:3, 8:2, or 9:1, and there are also a few using the ratio of 15:1. Eighty corn root images were taken as the test set and 600 corn root images were used as the training set. The new model is further trained by the previous training model, and the model converges in about 15 rounds, greatly reducing the time consumed for training, the final stable effect of the model before and after improvement was recorded. Subsequently, 120 images were added to the training set each time, and the model was trained using the same method and recorded. The experimental results are shown in **Figure 13**.

**Figure 14:**
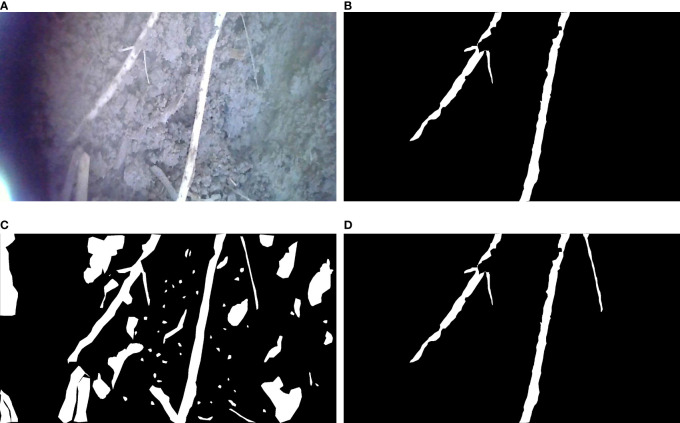
The segmentation effect before and after transfer learning. **(A)** In situ root system, **(B)** Labeled real msak map, **(C)** Segmentation effect without using transfer learning, **(D)** Segmentation effect aftr transferi learning.

From **Figure 13**, we can see that the training effect of the improved model is positively correlated with the number of training samples at the stage of the training set of 600 to 840 images, and there is little difference in the training effect of the model when the number of images exceeds 840. It proves that the improved model has good generalizability with sufficient data volume. The change in segmentation effect before and after transfer learning is shown in **Figure 14**, demonstrating that the network has good transfer learning performance.

## 4 Discussion

The condition of the plant root system is closely related to the growth condition of the plant, there are several research methods for the growth condition of the plant root system. The Minirhizotron method is a non-destructive research method that allows visual observation of root growth. Visually observing the root system from complex soils requires a lot of effort, so the complete root system needs to be separated from the soil to make the observation more direct and convenient.

The first requirement in the segmentation task is to obtain high-quality images that can successfully observe the root system. To maximize the quality of the observation images, the tubes are cleaned in advance, the observation tubes are buried in the soil months in advance, and the observation shots are taken using a side-by-side endoscope, which greatly prevents scratches and dust effects on the tube walls. In 2020, Xu et al. (2020) used U-Net to perform transfer learning on a dataset of tens of thousands of sheets and achieved satisfactory results. Shen et al. (2020) used the Deeplab-v3 network to segment the root system in homogeneous soil, but this method was too time-consuming. In this paper, we improve the U-Net network to improve its learning ability and conduct comparison experiments to verify that the improved network has improved the network learning ability while ensuring speed.

To further demonstrate the effectiveness of the network improvements in this paper, ablation experiments are conducted in this paper, and each step of improvement is added to the original network separately. The data are analyzed, and the effect of different improvements is recorded in detail in **Table 2**. To visualize the changes before and after the network improvement, the segmentation effect of the improved network is visualized (**Figure 9**), and the improved network can be intuitively felt to have a better segmentation effect on the edges and details of the image. Finally, in this paper, the improved network is tried to transfer learning and trained from scratch on different datasets, and the desired segmentation results are achieved (**Figure 13**). Compared with the traditional manual segmentation method, the segmentation time for a single image is reduced from tens of minutes to tens of seconds. This is a great time saving and we believe that the use of this method will greatly facilitate the study of root morphology. During the training process of the transfer learning experiment, we also found that increasing the amount of data does not enhance the segmentation effect. With 840 images as the training set, the training results of the network already tend to be optimal, and adding more data sets would be a waste of time.

In addition to the application of the Minirhizotron method, the newly developed model may be extended to medical, remote sensing, and unmanned vehicles. In the field of medicine, the segmentation of medical images can assist in determining human diseases and identifying the location of lesions, which will greatly improve medical efficiency. Wang et al. (2022) conducted a detailed survey on deep learning segmentation networks. Comparisons were made in terms of backbone network selection, network block design, and loss function improvement. Bhattarai et al. (2023) extended an existing semantic segmentation network, trained in a multi-task framework, and applied their studied network model to two challenging medical semantic segmentation datasets. In the field of remote sensing, statistics of land resources, smart agriculture and forestry, and environmental change monitoring can be realized. Wang et al. (2022) accomplished the semantic segmentation task of crop growth images in high-resolution agricultural remote sensing images, which can effectively improve agricultural intelligence. In the field of piloted driving, driverless technology for vehicles can be achieved by segmenting the scenes around the vehicle and planning them. Liu and Guan (2022) investigated pixel-level obstacle detection in complex driving scenarios, which meet the requirements of unmanned systems for obstacle detection accuracy.

Revisiting the whole experimental process, there are three parts worth discussing.

One is faster access to better-quality data sets. In the process of data collection, there is often a large amount of substandard data. Common substandard cases include blur caused by camera shake, obscuration by dust, reflections caused by light sources, etc. Therefore, a lot of screening is needed, and a lot of manpower is wasted. Secondly, we are looking for annotation tools with faster annotation speed. Before training starts, we need to annotate labels manually. Not only does it take a lot of time to annotate each image, but even the most experienced agroforestry experts will have some misjudgments about the root system and inevitably introduce errors. Faster annotation of labels will be needed in future studies. Third, the network model needs to be retrained each time, each training needs to consume a lot of time, and we still need to do a lot of work for fast application.

To address the above three issues, the following work can be done next. Firstly, for the acquisition of the dataset, considering that the most influential is the different root backgrounds, GAN networks can be used to generate different root backgrounds for data generation.2019, Tian et al. (2019) used CycleGAN to learn the characteristics of anthracnose apple images and transfer them into healthy apple images, using GAN networks will generate backgrounds with different textures, making the model more generalizable. Thus introducing GAN networks to generate different backgrounds can make the dataset adaptively data enhanced and get more generalizable experimental results. Secondly, in terms of annotation methods, we can choose not only the latest annotation tools but also pre-trained models or self-supervised learning models to assist in the annotation. Related work was carried out by Lin et al. (2016). Finally, we also provide three ideas for the problem of too long model training time. (a).Choosing the appropriate pre-trained model, which can reduce the number of model convergence rounds and achieve the desired at a faster speed. Han et al. (2021) have worked on the importance of pre-training models and how to train suitable pre-training models. (b). Selecting lightweight models for improvement to achieve the desired training effect, which is the idea used in this paper. (c). Using models of continuous learning, Hua et al. (2022) have proposed a continuous learning model without hyperparameters in the NLU domain, using old information and new information added continuously for learning, yielding more generalizable performance. Wang and Zhao (2022) made an exploration of continuous learning for tree species recognition, which to some extent solved the explosive forgetting problem of deep learning and made it possible to train a large range of data.

## Conclusion

In this paper, a trainable convolutional neural network method is proposed to improve the learning ability of the network by changing its structure. In the peanut segmentation task, the three evaluation metrics are pixel accuracy of 0.9917, Intersection Over Union of 0.9548, and F1-score of 95.10. The improved network is successfully demonstrated to have a good segmentation effect and learning ability. In addition, we use the transfer learning approach to test on different datasets, the test explores the data application strategy and proves the generalizability of the model. The improved network maintains a high segmentation level for different soil backgrounds and different crops, successfully demonstrating the good transferability of the improved network. Compared with manual methods, the proposed method in this paper can effectively improve the efficiency of root segmentation in soil and provide an effective aid for root segmentation tasks.

## Data availability statement

The raw data supporting the conclusions of this article will be made available by the authors, without undue reservation.

## Author contributions

YL and YH conceived the idea and proposed the method. YL, YH, and MW were involved in preparing the equipment and acquiring the data. YL and YH wrote the code and tested the method. YL wrote the manuscript. YL, YH, MW, and YZ revised the manuscript. All authors contributed to the article and approved the submitted version.
